# A seawater triggered dynamic coordinate bond and its application for underwater self-healing and reclaiming of lipophilic polymer†
†Electronic supplementary information (ESI) available: Route of synthesis, details of characterization methods, and results of structure and property measurements. See DOI: 10.1039/c5sc03483c


**DOI:** 10.1039/c5sc03483c

**Published:** 2016-01-12

**Authors:** Nan Nan Xia, Xiao Min Xiong, Junhu Wang, Min Zhi Rong, Ming Qiu Zhang

**Affiliations:** a Key Laboratory for Polymeric Composite and Functional Materials of Ministry of Education , GD HPPC Lab , School of Chemistry and Chemical Engineering , Sun Yat-Sen University , Guangzhou 510275 , China . Email: cesrmz@mail.sysu.edu.cn ; Email: ceszmq@mail.sysu.edu.cn; b School of Physics and Engineering , Sun Yat-Sen University , Guangzhou 510275 , China; c Mössbauer Effect Data Center & Laboratory of Catalysts and New Materials for Aerospace , Dalian Institute of Chemical Physics , Dalian 116023 , China

## Abstract

In this work, water triggered dynamic catechol–Fe^3+^ coordinate bonds are revealed and studied at atomic, molecular and macroscopic levels using Mössbauer spectroscopy, rheological analysis, *etc.* DOPA–iron complexation is found to be dynamic in the presence of water, and this dynamic manner is immobilized after removing water. Accordingly, a water saturated lipophilic polymer containing catechol–Fe^3+^ crosslinks, rather than the dry version, exhibits dynamic coordination–dissociation behavior. In addition, a migration of iron proves to be enabled in the catechol–Fe^3+^ crosslinked polymer immersed in seawater. Rearrangement of the dynamic catechol–Fe^3+^ coordinate bonds among different molecules is thus favored. Based on these results, we develop a bulk lipophilic polymer solid capable of repeated autonomic recovery of strength in seawater without manual intervention. When the polymer is damaged in seawater, reshuffling of the mobile hyperbranched polymer networks across the crack interface, owing to the dynamic catechol–Fe^3+^ crosslinkages activated by the alkaline circumstances, rebinds the damaged site. By taking advantage of the same mechanism, the polymer can be remolded with the help of seawater and this recycled polymer is still self-healable in seawater. Unlike in the case of conventional polymers where water would shield macromolecules from interacting, here, seawater is a necessary environmental assistant for the material interaction to take effect. The outcomes are beneficial for deepening the understanding of coordinate bonds, and the development of robust underwater self-healing lipophilic polymers.

## Abstract 2

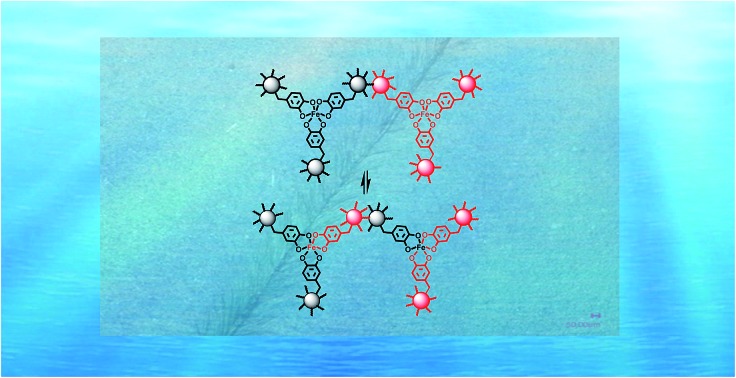
Bulk polymer capable of repeatedly underwater self-healing and reclaiming is synthesized under the inspiration of the formation of a mussel byssus cuticle.

## Introduction

The ever growing application of polymers and polymer composites in the marine and offshore industry has increased the demands on improving their reliability and durability. Imparting underwater self-healing capability to lipophilic bulk solid polymers is undoubtedly a fundamental solution to the problem. So far, however, very few studies are devoted to bulk polymer solids targeted for strength recovery underwater^1^ probably because of a lack of an appropriate healing strategy.^2^–^4^ In the case of extrinsic self-healing based on an embedded healing agent,^5^,^6^ for example, either the catalyst would be deactivated or the reaction of the released healing agent would be inhibited by water. With respect to intrinsic self-healing polymers utilizing reversible bonding,^7^ which are often lipophilic, the macromolecular chains on the cracked surface tend to shrink in water, preventing their diffusion and collision across the interface. Actually, the adhesion of hydrophobic polymers in water is quite difficult because of the very low van der Waals interaction energy derived from a significant decrease of the Hamaker constant in water, and the attenuation of electrostatic interactions induced by the water barrier layer. Although hydrogen bonds can be used to rebind a fractured surface in acidic media, water would preferentially form hydrogen bonds with macromolecules on the fractured surface (if any), and shield the macromolecules from their interaction.

It is worth noting that mussels have a superior ability to anchor firmly to all sorts of (wet) surfaces.^8^ What we learned from the animal is that 3,4-dihydroxyphenyl-l-alanine (DOPA) accounts for the water-resistant adhesive characteristics. Coordination between catechol groups of the dopamine and cations from seawater contributes to the hardness and extensibility of the cuticle of mussel byssal threads.^9^,^10^ However, the mechanism of this type of underwater stickiness has not yet been fully understood. Its potential application in the underwater self-healing of hydrophobic bulk polymer solid was not explored.

To transfer the functionality of the DOPA–metal interaction to synthetic polymers for self-healing in seawater, we have to first know (i) whether the coordination bond is dynamic and (ii) how to activate the dynamic behavior. These decide the structure of the envisaged polymer and the healing conditions as well. Only when the polymer is dynamically bonded, can the disconnected bonds be simultaneously recombined.^11^ As a result, crack healing is allowed to be completed in single step (*e.g.*, at constant temperature and/or pH), rather than following the two-step fashion of remending based on Diels–Alder bonds.^12^

As shown hereinafter, the catechol–Fe^3+^ coordinate bond is found to be dynamic in the presence of water, but remains static in a dry environment. This habit certainly favors the construction of an underwater self-healable polymer since water becomes an assistant of the healing chemistry. Accordingly, a seawater responsive polymer based on hyperbranched polyurethane (HBPU) with functional catechol and hydrophilic carboxyl end groups is designed (ESI, Fig. S1†). The catechol groups interact with Fe^3+^ forming a dynamic reversible catechol–Fe^3+^ coordinate bond to crosslink the polymer, and the carboxyl groups are responsible for slightly increasing the material's hydrophilicity. More importantly, the hyperbranched polymer ensures mobility of the networked structure, matching the dynamic catechol–Fe^3+^ bonds. When the polymer is damaged in seawater, rearrangement of the mobile hyperbranched polymer networks is stimulated across the fracture interface, which provides the polymer solid with both repeated autonomic strength restoration capability and reprocessability in seawater.

## Experimental

### Materials

Analytical grade polytetrahydrofuran (PTMEG, *M*_n_ ≈ 2000), isophorone diisocyanate (IPDI), dimethyl formamide (DMF), dimethylol propionic acid (DMPA), dibutyltin dilaurate (DBTDL), dopamine hydrochloride, ferric chloride, and triethylamine (TEA) were purchased from Sigma-Aldrich and used as received without further purification. Hydroxyl-terminated hyperbranched polyester (*n*_(–OH)_ = 12, *M*_n_ ≈ 1250) was purchased from Wuhan Hyperbranched Polymer Science & Technology Co., Ltd, China. PTMEG was dehydrated at 100 °C under vacuum for more than 24 h in advance.

### Synthesis of HBPU–DMPA–[Fe(DOPA)_3_] (ESI, Fig. S1†)

Polytetrahydrofuran (PTMEG, *M*_n_ ≈ 2000, 10.00 g, 0.005 mol) was dissolved in dimethyl formamide (DMF, 20 g), and then isophorone diisocyanate (IPDI, 2.22 g, 0.01 mol) dissolved in DMF (30 g) was added in a dry nitrogen atmosphere at 60 °C. The feed mole ratio was *n*_PTMEG_ : *n*_IPDI_ = 1 : 2. Under the catalysis of two drops of dibutyltin dilaurate (DBTDL), prepolymerization was carried out for 6 h under mechanical stirring. Afterwards, the hyperbranched polyester (HBPE, 0.520 g, 4.02 × 10^–4^ mol) dissolved in DMF (2 g) was incorporated into the prepolymerization solution at 40 °C with a feed mole ratio of *n*_HBPE_ : *n*_IPDI_ = 1.005 : 25 (*i.e.* the mole ratio of hydroxyl in HBPE to isocyanate = 1 : 2). Having reacted for 4 h under mechanical stirring, dimethylol propionic acid (DMPA, 0.335 g, 0.0025 mol) dissolved in DMF (1 g) was added (*n*_DMPA_ : *n*_IPDI_ = 1 : 4). The reaction was allowed to proceed for 4 h, and then the system was cooled down to 20 °C. Dopamine hydrochloride (0.474 g, 0.0025 mol) in DMF (28 g) was added with a feed mole ratio of *n*_DOPA_ : *n*_IPDI_ = 1 : 8, which was followed by a dropwise injection of triethylamine (TEA) to neutralize the hydrochloric acid. The reaction of chain extension was carried out for 10 h. Finally, FeCl_3_ (0.135 g, 8.32 × 10^–4^ mol) in DMF was added at an Fe^3+^/dopamine molar ratio of 1 : 3, and TEA was used to adjust the pH of the reaction system to about 9. To prepare a reference polymer for comparison, HBPU–DMPA–phenylethylamine was synthesized following the above procedures for HBPU–DMPA–DOPA, except that DOPA was replaced by phenylethylamine which resembles DOPA but excludes catechol groups.

To reveal the polymer microstructure, the intermediate HBPU–DMPA–DOPA was verified by Fourier transform infrared (FTIR, ESI, Fig. S2†) and nuclear magnetic resonance (NMR) spectroscopy (ESI, Fig. S3†). The content of dopamine in the polymer was determined by ultraviolet-visible (UV-vis) spectroscopy (ESI, Fig. S4†), and approaches the theoretical value. Moreover, the dynamic mechanical behavior of the end product HBPU–DMPA–[Fe(DOPA)_3_] was characterized (ESI, Fig. S5†) to perceive its *T*_g_. Meanwhile, electron paramagnetic resonance (EPR) spectroscopy was applied to study the change of crosslink status in HBPU–DMPA–[Fe(DOPA)_3_] with pH (ESI, Fig. S6†). Before the measurements, the polymer was immersed in water with different pH values and vacuum dried. In the EPR spectra of HBPU–DMPA–[Fe(DOPA)_3_], there are two peaks assigned to high-spin Fe^3+^ centers (*g* = 4.344, 1553 G) and organic radicals (*g* = 2.015, 3348 G),^13^ which are absent in the spectrum of HBPU–DMPA–DOPA. The later signal represents iron ions in a tris coordination environment. With a decreasing pH of the immersion water, the peak height at 3348 G decreases owing to a reduction of Fe^3+^. Mono- or bis-catechol–Fe^3+^ crosslinks appear in the polymer network accordingly.2c,^13^ The deduction of coordination number is supported by the observations of Raman spectroscopy as follows (ESI, Fig. S7†). Differences in the band at 500–650 cm^–1^ originating from the chelation of Fe^3+^ with oxygen atoms of catechol^14^ exist between the same group of samples. The peak intensity also decreases with decreasing pH of the immersion water. This agrees with the aforesaid transition from tris- to bis- and mono-coordinated Fe^3+^ species.

## Results and discussion

Earlier studies have found that DOPA could chelate with metal ions forming coordination bonds. However, whether these bonds are dynamically reversible in dry or wet circumstances has not been revealed. In fact, the coordinate bond is a kind of 2-center, 2-electron covalent bond in which the two electrons derive from the same atom. But in all cases the bond is a covalent one, and the prefix coordinate merely serves to indicate the origin of the electrons used in creating the bond. To have a fundamental understanding of the dynamic features of DOPA–Fe^3+^ coordination bonds at an atomic level, room temperature ^57^Fe Mössbauer measurements of a model complex of Fe[DOPA]_*n*_ (*n* = 1, 2, 3) were performed in dry and water saturated samples, because (i) water is likely to affect the iron coordination environment and symmetry of the electron cloud at the Fe^3+^ position, and (ii) the target polymer is planned to be self-healable underwater.

It is observed from Table 1 and ESI, Fig. S8† that a single state of iron exists in water saturated samples, while two states of iron are always detected in the anhydrous samples. This proves that the DOPA–iron complexation becomes dynamic in the presence of water and the dynamic manner is immobilized after removing water. Under the conditions of pH = 9, the Mössbauer parameters of anhydrous Fe[DOPA]_3_ indicate the existence of tris- and bis-coordination bonds. When the sample was saturated with water, tris-coordination bonds play the main role due to the dynamic coordination–dissociation of the DOPA–iron interaction. At pH = 7, the Mössbauer parameters of the anhydrous Fe[DOPA]_2_ resemble those of the water saturated version, and only a single state of iron appears in both samples. Clearly, the dynamic resonated bis-coordination between DOPA and Fe^3+^ in the water saturated sample behaves like the static coordination in the anhydrous sample. When the pH is reduced to 4, two types of resonance signals appear again on the Mössbauer spectrum of the anhydrous Fe[DOPA], and a single type of signal is perceived in the water saturated sample because of the dynamic nature of the coordination bonds.

**Table 1:** Room temperature ^57^Fe Mössbauer parameters obtained by fitting the spectra in ESI, Fig. S8

Samples	Isomer shift (mm s^–1^)	Quadrupole splitting (mm s^–1^)	Relative component area (%)
Fe[DOPA]_3_ (pH = 9)	0.40	0.89	78
	0.13	0.55	22
Water saturated Fe[DOPA]_3_ (pH = 9)	0.37	0.87	100
Fe[DOPA]_2_ (pH = 7)	0.43	0.78	100
Water saturated Fe[DOPA]_2_ (pH = 7)	0.43	0.78	100
Fe[DOPA] (pH = 4)	0.41	0.81	91
	1.29	2.93	9
Water saturated Fe[DOPA] (pH = 4)	0.40	0.82	100

On the basis of the above investigation, we further examined whether such a dynamic feature is inherited by the catechol–Fe^3+^ coordinate bond in polymers. If so, the polymer HBPU–DMPA–[Fe(DOPA)_3_] that is crosslinked by catechol–Fe^3+^ bonds should enable the rearrangement or reshuffling of the networks upon triggering by water. The rheological spectra of the specimens demonstrate that this is the case (ESI, Fig. S9a†). The frequency dependence of the storage shear modulus, *G*′, of the water saturated specimen intersects with that of the loss shear modulus, *G*′′, at a low frequency regime. This means that the material is changed from elastic-like (*G*′ > *G*′′) to viscous-like (*G*′ < *G*′′) with decreasing frequency, because the disconnected networks need time to be reconnected.^15^ Comparatively, this is not observed in the dry specimen (ESI, Fig. S9b†), highlighting the indispensable role of the water stimulus. This means that dynamic coordination–dissociation of catechol–Fe^3+^ crosslinks only takes place in water. Besides, for the system without Fe^3+^, the polymer remains linear, so that network reconfiguration is also unavailable (ESI, Fig. S9c†).

**Fig. 1:**
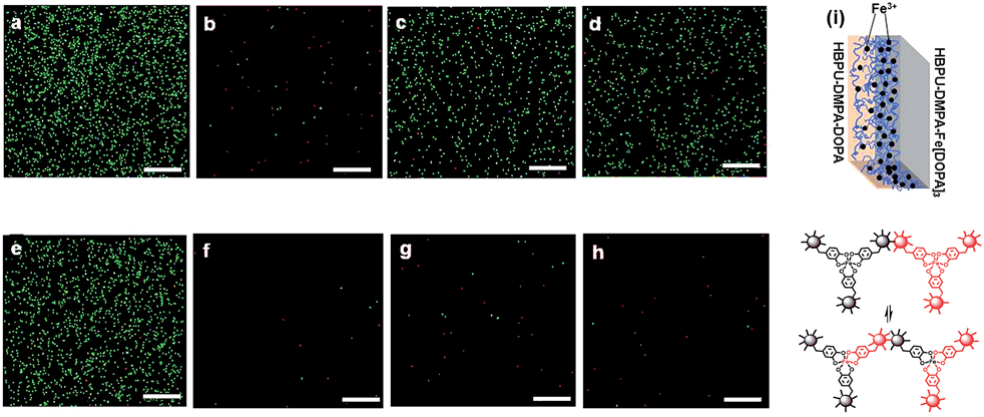
X-ray energy dispersive spectroscopy (EDS) analysis, with iron as the indicator element, of the outside surfaces of binary polymer films joined in artificial seawater (pH = 8.3), which does not contain any iron.^16^ The attached scale bars represent 100 μm in length. (a) Outside surface of HBPU–DMPA–[Fe(DOPA)_3_] of the binary films of HBPU–DMPA–[Fe(DOPA)_3_]/HBPU–DMPA–DOPA; time of joining: 0 h. (b)–(d) Outside surface of HBPU–DMPA–DOPA of the binary films of HBPU–DMPA–[Fe(DOPA)_3_]/HBPU–DMPA–DOPA; time of joining: (b) 0 h, (c) 24 h and (d) 48 h. (e) Outside surface of HBPU–DMPA–[Fe(DOPA)_3_] of the binary films of HBPU–DMPA–[Fe(DOPA)_3_]/HBPU–DMPA–phenethylamine; time of joining: 0 h. (f)–(h) Outside surface of HBPU–DMPA–phenethylamine of the binary films of HBPU–DMPA–[Fe(DOPA)_3_]/HBPU–DMPA–phenethylamine; time of joining: (f) 0 h, (g) 24 h and (h) 48 h. (i) Diagram showing the migration of Fe^3+^ from HBPU–DMPA–[Fe(DOPA)_3_] to HBPU–DMPA–DOPA and the corresponding dynamic catechol–Fe^3+^ bonds.

For the purposes of rebinding a cracked polymer with dynamic catechol–Fe^3+^ crosslinks, the iron ions should also be mobile throughout the material in cooperation with the network rearrangement. Otherwise, the reformed crosslinks have to be localized and less efficiently bridge the crack interface for healing. The migration ability of Fe^3+^ was verified by closely joining HBPU–DMPA–[Fe(DOPA)_3_] with HBPU–DMPA–DOPA which contains DOPA without iron. After immersion of the two joined films in artificial seawater for a certain time, iron ions appeared on the surface of the latter (Fig. 1a–d). For comparison, the DOPA in HBPU–DMPA–DOPA was replaced by phenethylamine which has a similar structure to DOPA but cannot bond to Fe^3+^ due to a lack of catechol groups. No migration of Fe^3+^ occurs at the interface of HBPU–DMPA–[Fe(DOPA)_3_]/HBPU–DMPA–phenethylamine under the same conditions (Fig. 1e–h). Furthermore, we also found that Fe^3+^ cannot travel from HBPU–DMPA–[Fe(DOPA)_3_] to HBPU–DMPA–DOPA in the absence of seawater. Evidently, the migration of Fe^3+^ is not driven by the Fe^3+^ concentration difference and sample polarity difference (ESI, Fig. S10†), but by the dynamic bonding of catechol–Fe^3+^ complexation.

**Fig. 2:**
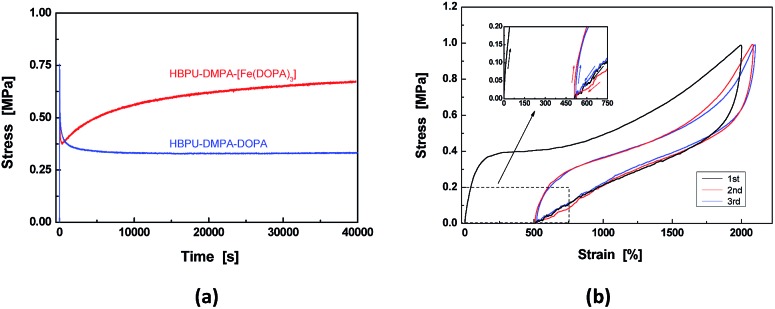
(a) Stress-time curves of HBPU–DMPA–[Fe(DOPA)_3_] and HBPU–DMPA–DOPA measured during tensile stress relaxation tests. Both the specimens were saturated by water at pH = 9; (b) tensile stress–strain curves of HBPU–DMPA–[Fe(DOPA)_3_] recorded during cyclic loading and unloading tests beyond the yield region in water (pH = 9). Arrows indicate the directions of loading and unloading. Only cycles 1, 2 and 3 are shown for clarity (cycles 4 and 5 are not given). The cyclic tests were continuously conducted without resting between successive cycles. Test temperature: 25 °C.

The dynamic catechol–Fe^3+^ bonds coupled with mobile Fe^3+^ imply that the crosslinkages of HBPU–DMPA–[Fe(DOPA)_3_] are dynamic in seawater, which exerts obvious influences on the macroscopic mechanical properties of the polymer. Fig. 2a depicts typical tensile stress relaxation behaviors of the related materials. Normally, dynamic reversible bonds may lead to almost complete stress relaxation of the polymer because the crosslinked networks are allowed to be rearranged to a less stretched state owing to bond fission/recombination.15a,^17^ However, the network reshuffling of HBPU–DMPA–[Fe(DOPA)_3_] develops so fast that the stress increases with time after the initial drop due to the conformation change (Fig. 2a). The formation of transient crosslinking among neighbor macromolecules accompanying a Poisson contraction induced a reduction of the intermolecular distance in lateral direction that should account for the phenomenon. As for the control HBPU–DMPA–DOPA, this performs like other irreversibly bonded polymers. The stress rapidly declines to a stable value and then no longer changes with time.

Meanwhile, the cyclic tensile tests also show that for HBPU–DMPA–[Fe(DOPA)_3_], except the first hysteresis loop due to permanent plastic deformation, the rest hysteresis loops almost overlap each other without residual strain (Fig. 2b). The dynamic bonding of catechol–Fe^3+^ complexation must have helped to re-establish and rearrange the crosslinks during unloading. In contrast, residual strains are found after the second and third cycles in the control HBPU–DMPA–DOPA and the corresponding hysteresis loops do not overlap (ESI, Fig. S11†).

Since the above experiments have disclosed the water triggered dynamic feature of catechol–Fe^3+^ bonds, HBPU–DMPA–[Fe(DOPA)_3_] is expected to be able to self-heal underwater. Two methods were applied for the evaluation. Firstly, a HBPU–DMPA–[Fe(DOPA)_3_] film sample was cut in artificial seawater^16^ (pH = 8.3) at 25 °C and then the damaged sample was allowed to stay in the water for healing (ESI, Fig. S12†). After 24 h, the wound was healed and the second cut was made across the trace of the first one. Similarly, it was also healed in artificial seawater. The disappearance of the intersection, which had been subjected to two cut–repair cycles, reveals the repeatable healability of the material. In contrast, the control failed to heal the wounds.

**Fig. 3:**
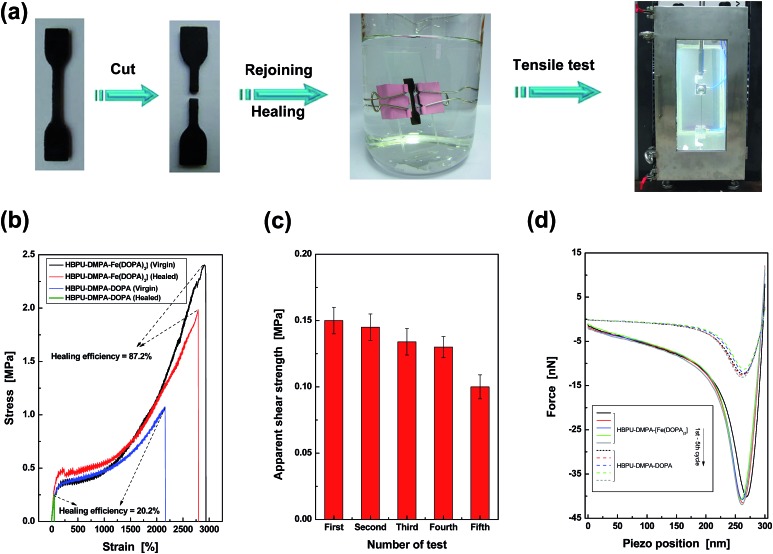
Quantitative evaluation of self-healing capability of HBPU–DMPA–[Fe(DOPA)_3_]. (a) Dumbbell specimen was cut, recombined, healed, and tested to failure under tension. The entire procedures were conducted in artificial seawater at 25 °C. (b) Typical tensile stress–strain curves of HBPU–DMPA–[Fe(DOPA)_3_] and the control HBPU–DMPA–DOPA. The healing efficiencies were calculated by strength recovery (refer to the section entitled “Characterization” in the ESI† for more details). If the healing efficiencies are calculated by comparing the energies absorbed (*i.e.* the area under the stress–strain curves) of healed and virgin specimens, the values are 92.5% for HBPU–DMPA–[Fe(DOPA)_3_] and 2.5% for the control HBPU–DMPA–DOPA. (c) Apparent shear strength of HBPU–DMPA–[Fe(DOPA)_3_] measured by multiple lap shear tests in artificial seawater at 25 °C. (d) Typical AFM pull-off curves recorded during repeated approach and retraction of probes. The probes were functionalized by the same polymer as the substrate material to be tested. Both the polymers were saturated by water at pH = 9.

Secondly, a dumbbell specimen was cut into two pieces in artificial seawater (Fig. 3a). Afterwards, the broken surfaces were carefully brought into contact, and the recombined specimen was clamped by two pieces of glass slide for 24 h. The entire process was conducted in artificial seawater at 25 °C. Finally, the healed specimen was subjected to tensile testing in a chamber full of artificial seawater. The healing efficiency was calculated from a tensile strength ratio of healed and virgin specimens. By comparing to the negligible healing effect of the control HBPU–DMPA–DOPA, which lacks catechol–Fe^3+^ crosslinks, it is shown that HBPU–DMPA–[Fe(DOPA)_3_] can indeed self-heal in seawater with a satisfactory degree of strength recovery (Fig. 3b). The catechol–Fe^3+^ dynamic crosslinks must have made the predominant contribution. Such crack remending is completed free of manual intervention (like tuning environmental pH,2c,^10^ pretreatment in buffer^1^ or driving water away from the rejoined region). Furthermore, Fig. 3b exhibits that HBPU–DMPA–[Fe(DOPA)_3_] has an extremely high failure strain (∼2900%) in addition to a moderate tensile strength (∼2.5 MPa). This should be attributed to the unique conformation of the hyperbranched polymer, which has less entanglement than traditional linear macromolecules and facilitates slippage among the chain segments.

Considering the fact that the broken parts of the tensile specimen are severely deformed after the large elongation, the repeated self-healability of the material can not be examined by recombining the fractured versions. Lap shear tests of iron plates bonded by HBPU–DMPA–[Fe(DOPA)_3_] were carried out in artificial seawater instead. After the first failure, the two iron plates were re-bonded and tested in seawater again. The results of such cyclic experiments indicate that the failure type is always cohesive (ESI, Fig. S14†), and the polymer has the capability of repeated self-healing in seawater (Fig. 3c).

Although the lap shear test led to a less severe deformation of the material than the tensile test, the fractured surfaces were still uneven, which cannot be flattened by the mild pressure applied for keeping apparent contact between the broken specimens during healing. With increasing the number of tests, more and more non-contactable regions appear, which reduces the effective healing at the interface so that the measured shear strength decreases accordingly. Nevertheless, the phenomenon does not perfectly reflect the nature of the material. The similar AFM pull-off forces confirm the reversibility of self-healing (Fig. 3d and ESI, Table S1†).

**Fig. 4:**
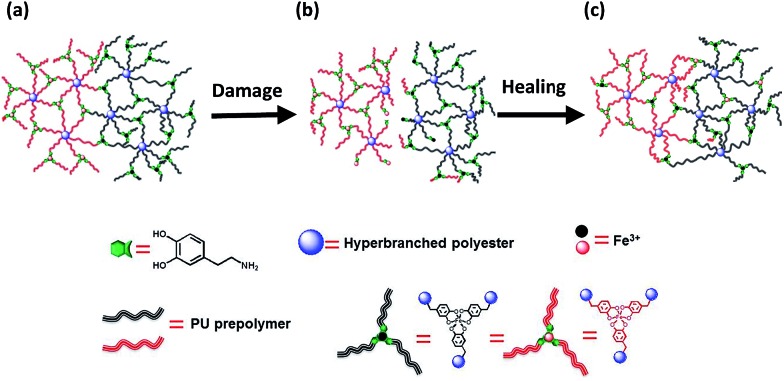
Rehabilitation of damaged HBPU–DMPA–[Fe(DOPA)_3_] in seawater. (a) Virgin polymer network. (b) Damaged polymer network containing ruptured (i) covalent bonds and (ii) catechol–Fe^3+^ crosslinks at the interface. (c) Reconnection of the damaged polymer network through formation of catechol–Fe^3+^ bonds at the interface with the aid of dynamic catechol–iron interactions. Note: the iron ions and macromolecules are color coded in this way (i) to distinguish the substances located at different sides of the material, and (ii) to highlight the dynamic rearrangement of the damaged networks during healing with the aid of the mobile iron ions.

As for the mechanism involved in the healing action, reformation of the ruptured catechol–Fe^3+^ coordination bonds and generation of new catechol–Fe^3+^ interaction across the crack interface should take responsibility (Fig. 4). Although the resulting polymer is generally lipophilic, the hydrophilicity of its damaged surface would be slightly improved due to the exposure of more carboxyl groups under the inducement of water (ESI, Fig. S10 and S15†), which benefits an interfacial inter-diffusion of macromolecules in water. Moreover, the carboxyl groups also facilitate the permeation of water molecules into the subsurface, bringing about the alkaline circumstances required for establishing catechol–Fe^3+^ coordination bonds. Whenever the polymer is damaged, spontaneous dynamic complexation between catechols and Fe^3+^ starts to reconnect the cracked parts in water, without the fear of a shielding effect of water. Ceylan *et al.* suggested that ferric ions diffused in and rebound to the peptide network crosslinked by peptide–iron complexation in a highly reversible fashion,2*o* while they did not provide experimental evidence. Our results demonstrate that Fe^3+^ can indeed move around in the polymer immersed in seawater (Fig. 1), which would lead to creation of delocalized catechol–Fe^3+^ crosslinks in cooperation with the dynamic complexation effect (Fig. 1i). Accordingly, a rearrangement of the network through the catechol–Fe^3+^ dynamic crosslinks gradually reconnects the damaged parts and restores the mechanical properties of the broken specimen. Besides, the catechol–Fe^3+^ complexation can effectively prevent DOPA from oxidation (ESI, Fig. S16†).

**Fig. 5:**
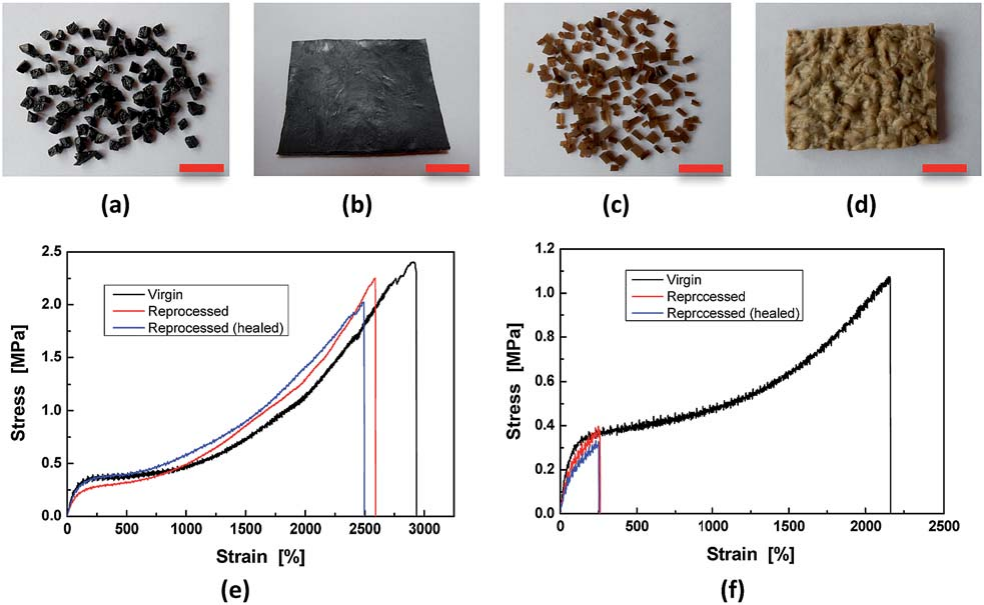
Reclaiming of the polymers. ((a), (b) and (e)) HBPU–DMPA–[Fe(DOPA)_3_]. ((c), (d) and (f)) HBPU–DMPA–DOPA. The scale bars represent 1 cm in length.

By making use of the seawater triggered dynamic catechol–Fe^3+^ coordination bonds, the crosslinked HBPU–DMPA–[Fe(DOPA)_3_] can even be reclaimed in seawater (Fig. 5). In this situation, sheeted HBPU–DMPA–[Fe(DOPA)_3_] was firstly cut into small pieces (Fig. 5a), which were then soaked in water of pH = 4 for 24 h to partly dissociate Fe(DOPA)_3_ to Fe(DOPA). Afterwards, the fragments were transferred to artificial seawater for 24 h to rebuild the tris-coordinated catechol–Fe^3+^ bonds especially across the fragments. Finally, the wet fragments were compression molded at 6 MPa for 48 h at room temperature to yield a sheet again (Fig. 5b). The reprocessed HBPU–DMPA–[Fe(DOPA)_3_] possesses strength similar to the original version, and is also self-healable in seawater (Fig. 5e). HBPU–DMPA–DOPA was reprocessed under the same conditions as HBPU–DMPA–[Fe(DOPA)_3_], but the resultant sheet is quite uneven (Fig. 5d) because the fragments (Fig. 5c) were poorly attached to one another. Comparatively, the reprocessed HBPU–DMPA–DOPA cannot recover its strength and the healing efficiency is rather low (Fig. 5f). Actually, the fragmented HBPU–DMPA–[Fe(DOPA)_3_] and HBPU–DMPA–DOPA have rather high specific surface areas, so their contact probability with water is also rather high. If the dynamic catechol–Fe^3+^ coordination bonds did not take effect, they would not be recombined into a compact bulk solid. Hence the result in Fig. 5 confirms the mechanism of seawater triggered dynamic coordination bonds from another aspect.

## Conclusions

We have proven that dynamic coordination–dissociation of catechol–Fe^3+^ coordination bonds can be carried out in the presence of water. On the basis of this finding, we further proposed an innovative concept of material design for the preparation of underwater self-healing polymers. Lipophilic hyperbranched polymer plays the role of a matrix, in which terminals are functionalized by dopamine and hydrophilic carboxyl groups, and then crosslinked by catechol–Fe^3+^ complexation. Hydrophilic modification of the macromolecules ensures interfacial chain diffusion and collision in water, while the alkaline seawater triggered dynamic reversible catechol–Fe^3+^ bonds lead to the rearrangement of polymer networks. By taking advantage of the specific composition and microstructure, the resultant bulk material is capable of repeatedly underwater self-healing and reclaiming. Despite the material not yet being optimized, the outcomes of this work open a new avenue to develop robust underwater self-healing polymers useful for marine engineering.

## Supplementary Material

Supplementary information
