# Supplementary material for: A seawater triggered dynamic coordinate bond and its application for underwater self-healing and reclaiming of lipophilic polymer
Source: Chem Sci. 2016 Jan 12;7(4):2736–42. doi: 10.1039/c5sc03483c (PMC5477145; doi:10.1039/c5sc03483c)
Supplement: Supplementary file 1 [file SC-007-C5SC03483C-s001.pdf]

Electronic Supplementary Material (ESI) for Chemical Science.
This journal is © The Royal Society of Chemistry 2016

Electronic Supplementary Information

Seawater Triggered Dynamic Coordinate Bond and Its Application to Underwater Self-healing and Reclaiming of Lipophilic Polymer

Nan Nan Xia,[†] Xiao Min Xiong,[‡] Junhu Wang,[§] Min Zhi Rong,*[,†] and Ming Qiu Zhang*[,†]

[†]*Key Laboratory for Polymeric Composite and Functional Materials of Ministry of Education, GD HPPC Lab, School of Chemistry and Chemical Engineering, Sun Yat-Sen University, Guangzhou 510275, China*
[‡]*School of Physics and Engineering, Sun Yat-Sen University, Guangzhou 510275, China*
[§]*Mössbauer Effect Data Center & Laboratory of Catalysts and New Materials for Aerospace, Dalian Institute of Chemical Physics, Dalian 116023, China*

Synthesis

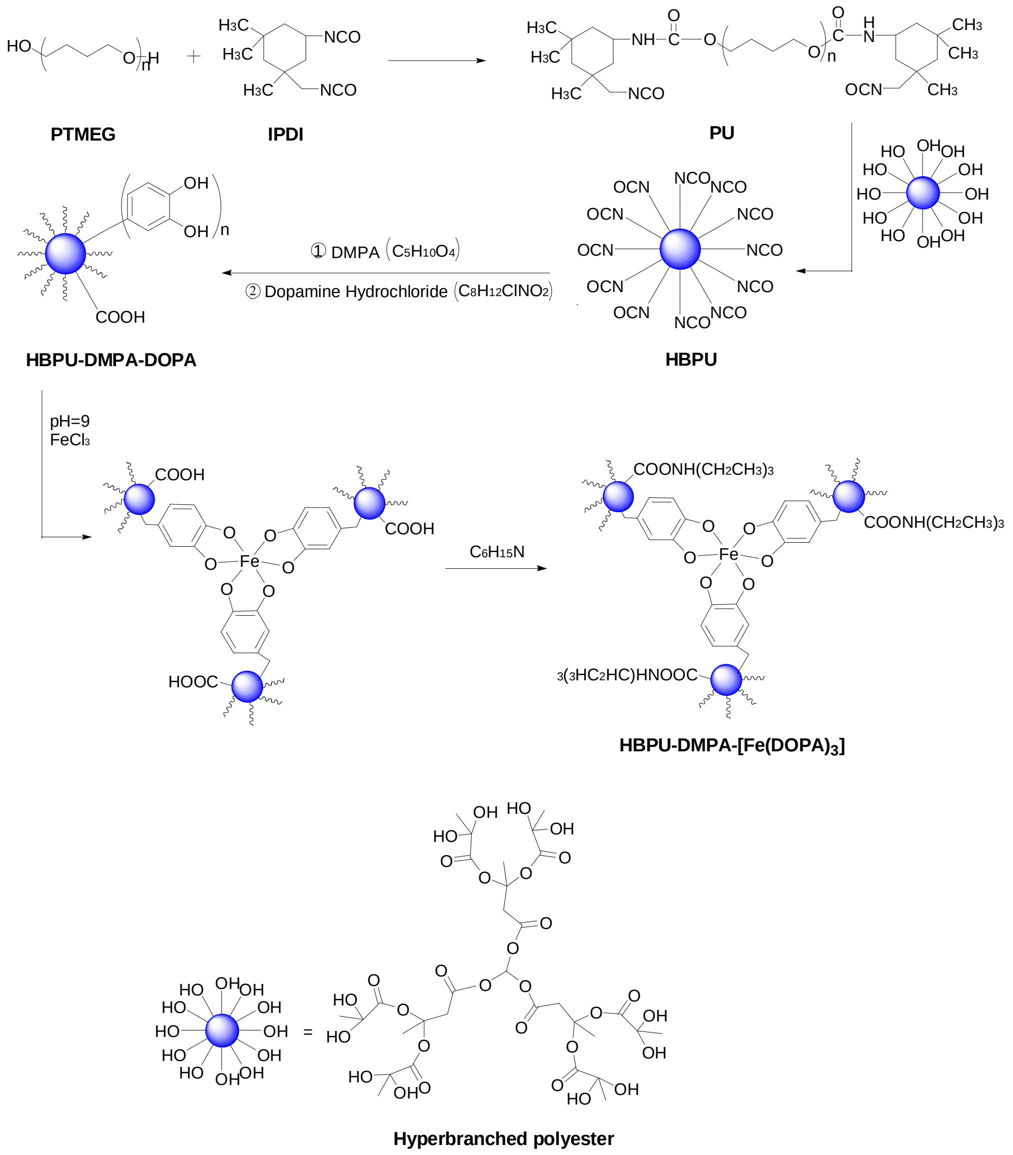

Figure S1. Synthesis of HBPU-DMPA-[Fe(DOPA)$_3$].

Characterization

Fourier transform infrared spectra were recorded using a Bruker EQUINOX55 FTIR spectrophotometer between 400 and 4000 cm^{-1} with a resolution of 4 cm^{-1} by the KBr sample holder method. Attenuated total reflectance (ATR) spectra were collected with a Bruker TENSOR 27 spectrophotometer between 1000 and 2000 cm^{-1} with a resolution of 4 cm^{-1}.

Proton nuclear magnetic resonance spectrum (^{1}H NMR) was obtained by using DMF-d_7 as the solvent on a Bruker AVANCE III spectrometer (400 MHz). The spectra were internally referenced to tetramethylsilane (TMS) standard.

Ultraviolet-visible (UV-Vis) spectra were measured by a Perkin Elmer Lambda 750 UV-Vis-NIR spectrophotometer.

Water contact angle was measured with a Krüss DSA 100 apparatus at ambient temperature. All the measurements were taken after a 3 μL water droplet was placed on the sample for about 1 min.

Evaluation of self-healing performance in seawater is described as follows. The synthesized polymer was filled in a silicone mold, and dried under vacuum at 40 °C for 48 h for the subsequent characterization. In the case of visual inspection, the sample (2 mm thick) was cut by a razor in artificial seawater at 25 °C to produce a scratch 20~50 μm wide, and then, the damaged film was allowed to stay in the water for healing. Photos recording change of the wound were taken by a camera attached to an optical microscope. As for quantitative assessment, underwater tensile test was performed on dumbbell-shaped specimen cut from the polymer film (2 mm thick) according to ISO 527-3 at a crosshead speed of 500 mm min^{-1} with a LWK-5 universal tester. Firstly, the specimen was bisected in artificial seawater, and then the broken parts were recombined under a gentle pressure of about 0.01 MPa in the water at 25 °C for healing. After 24 h, the healed specimen was tested in a chamber full of artificial seawater. The strength ratio of the healed specimen to the virgin specimen gives the measure of healing efficiency. Additionally, healing efficiency can also be calculated from the energies absorbed (i.e. the area under the tensile stress-strain curves) of healed and virgin specimens. To further understand the repeated healability of HBPU-DMPA-[Fe(DOPA)$_3$], the synthesized product was pasted onto iron plates, which were then bonded together with an adhered size of 25 mm × 12.5 mm under a pressure of 0.15 MPa, dried at 40 °C for 48 h in vacuum, and immersed in artificial seawater at 25 °C for 24 h. The lap shear test was conducted at a crosshead speed of 5 mm min^{-1} in water. The failed specimen was recombined in water under the pressure of 0.15 MPa for 24 h, and tested to failure in water again.

Electron paramagnetic resonance (EPR) spectra were recorded by a Brückner A300-10-12 EPR spectrometer. All data presented herein were taken at -70 °C. Bridge frequency was tuned to 9.45 GHz with a microwave power of 2.12 mW. Most

experiments were run with a sweep width of 6000 G, centered at 3480 G. When looking at the organic radical region, a sweep centered at 3348 G was used. The modulation amplitude was 1.00 G. To get EPR spectra of HBPU-DMPA-[Fe(DOPA)$_3$] complexes at different pH values, the polymers were respectively immersed in aqueous solution with pH = 4, 7 and 9 in advance, and dried by vacuum freeze drier for 2 days.

Raman spectra were collected by a confocal Raman microscope (Renishaw inVia). The diode-pumped 785 nm near infrared laser excitation was used in combination with a 20× microscope objective. The spectra were acquired using an air-cooled CCD behind a grating (300 g mm^{-1}) spectrograph with a spectral resolution of 4 cm^{-1}. Because the samples were sensitive to burning by the laser beam, laser power of 10 mW and exposure time of 20 s were used for all measurements. Each collected spectrum consisted of 60 accumulations of a 0.2 s integration time. For each sample, three spectra were collected from different regions and averaged.

Similarly, to get Raman spectra of HBPU-DMPA-[Fe(DOPA)$_3$] complexes at different pH values, HBPU-DMPA-[Fe(DOPA)$_3$] complexes were respectively immersed in aqueous solution with pH = 4, 7 and 9 in advance, and dried by vacuum freeze drier for 2 days.

To confirm the dynamic characteristics of catechol-Fe^{3+} bonds, HBPU-DMPA-[Fe(DOPA)$_3$] (30 μm thick) and HBPU-DMPA-DOPA (30 μm thick) were jointed in artificial seawater under a gentle pressure of 0.15 MPa at room temperature for 24 h to simulate the healing conditions of HBPU-DMPA-[Fe(DOPA)$_3$]. Then, the jointed binary films were vacuum dried and detected by X-ray energy dispersive spectroscopy (EDS). As a reference, jointed binary films of HBPU-DMPA-[Fe(DOPA)$_3$]/HBPU-DMPA-phenethylamine were prepared and tested in the same way as above.

Dynamic mechanical analysis (DMA) of film specimens (2 × 3 × 15 mm^3) was performed using a Mettler Toledo Instruments DMA SDTA861 in tensile mode under 0.5 Hz with a heating rate of 5 °C min^{-1} in nitrogen.

To measure pull-off force of a polymer (a measure of adhesion between the same polymers) using atomic force microscopy (AFM), the method suggested by Florin et al. was applied (refer to E.-L. Florin, V. T. Moy, H. E. Gaub, Adhesion forces between individual ligand-receptor pairs, Science, 1994, 264(15): 415). Firstly, glass

microsphere (50 μm in diameter) was coated by the polymer, and then glued to tipless AFM cantilever (Bruker, Model: TESP, Material: 0.01-0.025 Ohm-cm, Antimony (n) doped Si, spring constant: 20-80 N/m). Afterwards, the probe was brought into contact with the polymer film and retracted. All measurements were performed on the Bruker Multimode with ScanAsyst. Scanning rate: 1 Hz, scanning size: 5 nm, peak force setpoint: 77.16 nN, forward velocity: 414 nm s^{-1}, reverse velocity: 414 nm s^{-1}, contact time of the probe with the sample: 150 μs.

Rheological data were obtained from a strain-controlled ARG2 rheometer with 25 mm parallel-plate geometry (disk-shaped specimens: 10 mm in diameter and 2 mm in thickness). Frequency sweeps at 0.2 % strain were conducted at different temperatures.

Stress relaxation was measured at 25 °C in tensile mode at a constant strain of 10 % with 01dB-MetraviB DMA-25N.

Cyclic tensile tests were conducted at 25 °C with a LWK-5 universal tester. Crosshead speed of loading is 100 mm min^{-1}, and that of unloading is 10 mm min^{-1}.

The Mössbauer measurements were performed with a Topologic 500A spectrometer and a proportional counter at room temperature. $^{57}Co(Rh)$ moving in a constant acceleration mode was used as the radioactive source. The Doppler velocity of the spectrometer was calibrated with α-Fe foils. The spectra were fitted with Lorentzian peaks using Moss Winn 3.0i, and the free recoil fraction was assumed to be the same for all iron species. Model compounds $Fe[DOPA]_n$ were prepared and characterized as follows to simulate the polymer HBPU-DMPA-$[Fe(DOPA)_3]$ of this work. Dopamine hydrochloride (DOPA, 1 g) was dissolved in 10 ml distilled water, and then anhydrous ferric chloride was added at mole ratios of nDOPA : $nFeCl_3$ = 3:1, 2:1 and 1:1, respectively. The complex of $Fe[DOPA]_3$, $Fe[DOPA]_2$ and Fe[DOPA] were yielded accordingly. Afterwards, the pH value of the solution was adjusted by NaOH and HCl under stirring to 9, 7 and 4, respectively. Finally, the system was dried under vacuum at 50 °C for completely volatilizing moisture, and the product was ground into fine powders for the subsequent measurement. For comparison, some samples were saturated by water and then measured by Mössbauer spectroscopy, while their pH values were kept unchanged.

Supplementary results

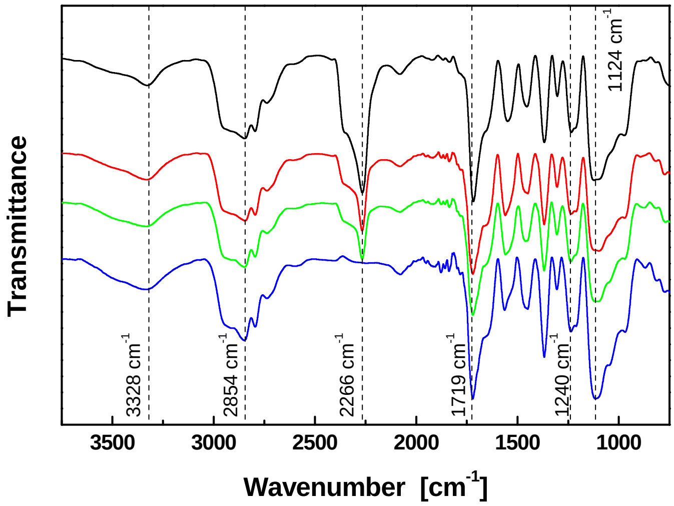

Figure S2. FTIR spectra of (a) PU (black curve), (b) HBPU (red curve), (c) HBPU-DMPA (green curve), and (d) HBPU-DMPA-DOPA (blue curve). The strong absorbance of PU at 3328, 2854 and 2266 cm^{-1} results from the stretching vibrations of –NH–, –$(CH_2)_2$- and –NCO, respectively. Meanwhile, the peaks at 1719, 1240 and 1124 cm^{-1} are attributed to the single bond vibrations of –COO– and –OH and fingerprint region of –CH–, respectively. Compared with the spectrum of PU, the absorbance between 3100 and 3600 cm^{-1} assigned to O–H stretching on the spectrum of HBPU is broader and the peak at 2266 cm^{-1} decreases. It proves the existence of hyperbranched polyester because its hydroxyl was not completely consumed during the reaction with PU and the amount of –NCO on PU was reduced after the reaction with the hyperbranched polyester. As for the spectrum of HBPU-DMPA, the peak at 2266 cm^{-1} keeps on decreasing in comparison with the spectrum of HBPU. This is due to the fact that DMPA has reacted with –NCO of the polymer. In the case of HBPU-DMPA-DOPA, although the peaks of O–H and N–H stretching overlap at 3100 ~ 3600 cm^{-1}, which are hard to be distinguished, the absence of the peak at 2266 cm^{-1} clearly manifests that the –NCO of the polymer has reacted with dopamine.

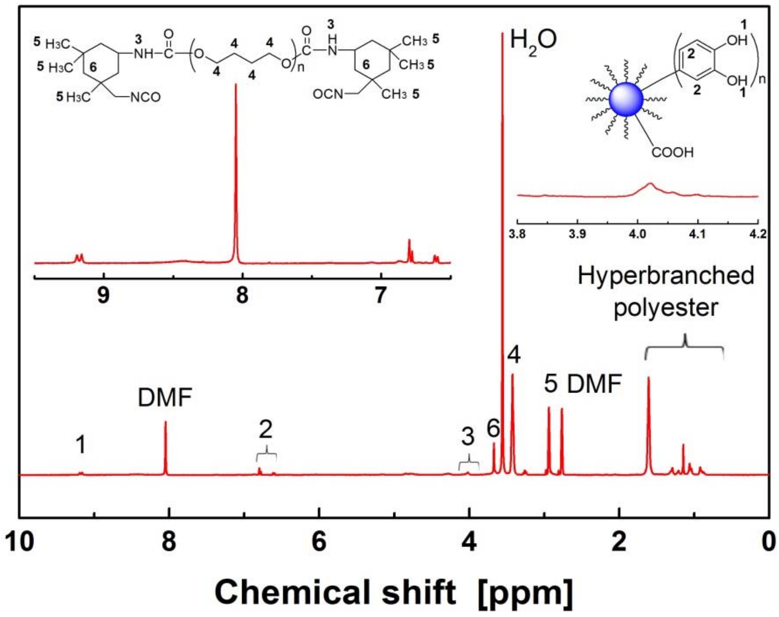

Figure S3. ^{1}H NMR spectrum of HBPU-DMPA-DOPA (9.16-9.19 (d, 2H, -OH of catechol), 6.59-6.68 (d, 3H, CH=CH of catechol), 4.02 (d, 1H, -NH- resulting from the reaction between catechol and IPDI), 3.42 and 3.85 (s, 4H, $-CH_2-CH_2-$ of dopamine, PTMEG and IPDI), 2.98-2.91 (d, 6H, $-CH_3$ of IPDI), 0.9-1.61 (hyperbranched polyester)). The results proved that dopamine has been bonded to the polymer chain.

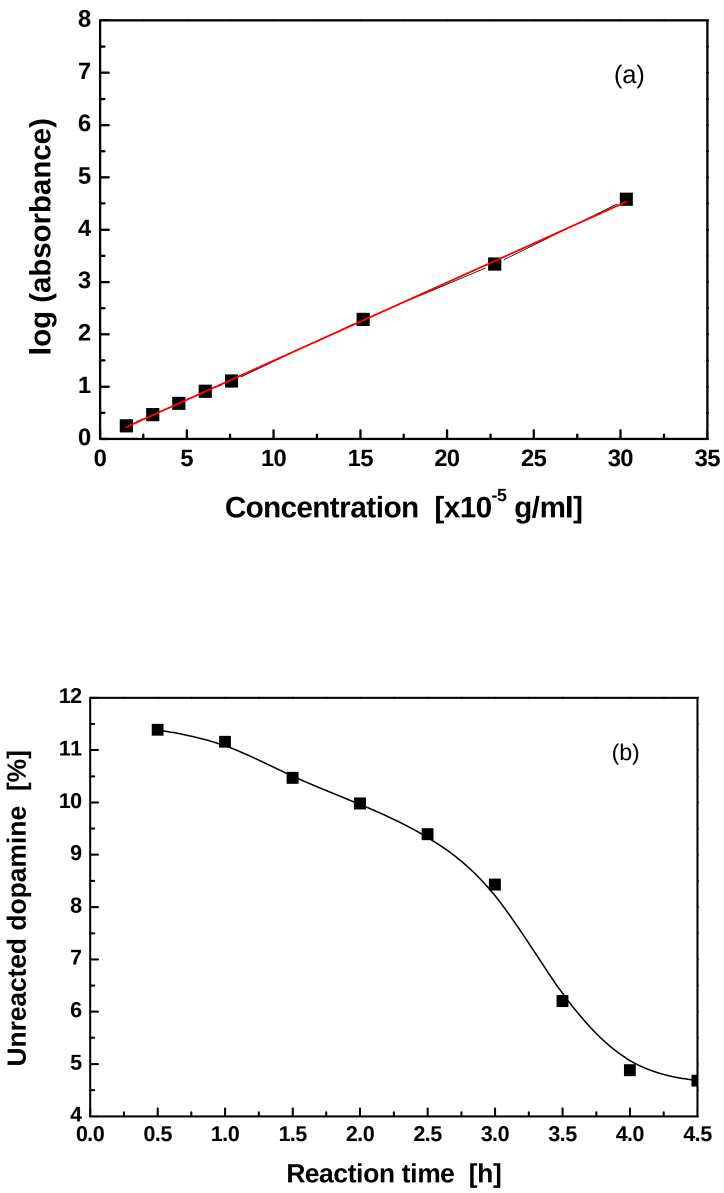

Figure S4. (a) Logarithmic absorbance at 280 nm of aqueous solution of dopamine versus concentration of dopamine. (b) Reaction time dependence of amount of unreacted dopamine in HBPU-DMPA-DOPA. The latter is characterized by the concentration of dopamine in water obtained by water extraction of HBPU-DMPA-DOPA.

As the absorbance at 280 nm on UV-Vis spectrum of aqueous solution of dopamine originates from catechol group, the peak height increases with a rise in

concentration of dopamine. Accordingly, a linear regression was made to correlate the dependence of the absorbance at 280 nm of aqueous solution of dopamine on concentration of dopamine (**Figure S4a**). By using the relationship shown in Figure S6a as a calibration curve, the content of dopamine in the polymer can be determined as follows. After the incorporation of dopamine into the reaction system, a certain amount of sample was taken out at set interval. Then, the sample was put into water for 24 h at room temperature, allowing completely dissolving out of the unreacted dopamine. Afterwards, the concentration of the unreacted dopamine in aqueous solution was quantified by UV-Vis spectroscopy with the aid of the plot in **Figure S4a**, and plotted as a function of reaction time (**Figure S4b**). When the amount of unreacted dopamine approached to a steady value, it means that the reaction is almost completed, so that the data at the reaction time of 4.5 h in **Figure S4b** can be used to estimate the ultimate content of dopamine in the polymer via the equation: $C_{DOPA} \cdot V_{H_2O}/(m_1 \cdot m_{DOPA}/m)$, where C_{DOPA} denotes the detected concentration of dopamine extracted in water, V_{H_2O} the volume of water, m_1 mass of the sampled polymer, m_{DOPA} mass of the dopamine fed to the reaction system, and m total mass of the reaction system. The content of dopamine in the polymer measured in this way is 3.29 %, very close to the theoretical value (3.50 %) calculated from the formulation.

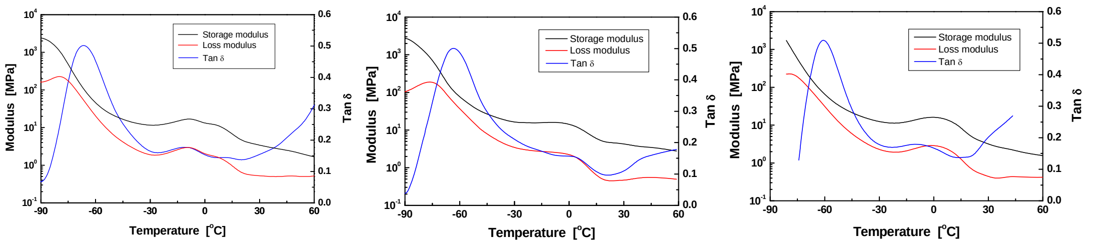

(a) (b) (c)

Figure S5. Temperature dependences of storage modulus, loss modulus and tan δ of (a) HBPU-DMPA-[Fe(DOPA)$_3$], (b) HBPU-DMPA-DOPA, and (c) HBPU-DMPA-phenethylamine. Frequency = 0.5 Hz.

The dynamic mechanical behaviors shown by the plots coincide with the common rule of polyurethane. In particular, two glass transition temperatures, T_g, which correspond to soft and hard segments of the polymers, respectively, are observed on each tan δ versus temperature curve. It is seen that T_g of the soft segments of HBPU-DMPA-[Fe(DOPA)$_3$] (-63.3 °C) is higher than those of HBPU-DMPA-DOPA (-66.0 °C) and HBPU-DMPA-phenethylamine (-68.5 °C). Meanwhile, T_g of the hard segments of HBPU-DMPA-DOPA (4.6 °C) is higher than HBPU-DMPA-phenethylamine (-6.7 °C) and HBPU-DMPA-[Fe(DOPA)$_3$] (-7.5 °C). Clearly, the appearance of catechol-Fe^{3+} complex crosslinks in the polyurethanes leads to shift of T_g of the soft segments of HBPU-DMPA-[Fe(DOPA)$_3$] to higher temperature regime, and shift of T_g of the hard segments of HBPU-DMPA-[Fe(DOPA)$_3$] to lower temperature regime. This should originate from disordering of the short-range order of the hard segments (refer to R. W. Seymour, S. L. Cooper, Thermal analysis of polyurethane block polymers, Macromolecules, 6, 1973, 48) due to the improved miscibility between the soft and hard segments after crosslinking.

As for the loss moduli of the polymers, their temperature dependences resemble those of tan δ as usual. On the other hand, the storage modulus of HBPU-DMPA-[Fe(DOPA)$_3$] at room temperature is about 3.9 MPa, which implies that the polymer has moderate stiffness for structural application.

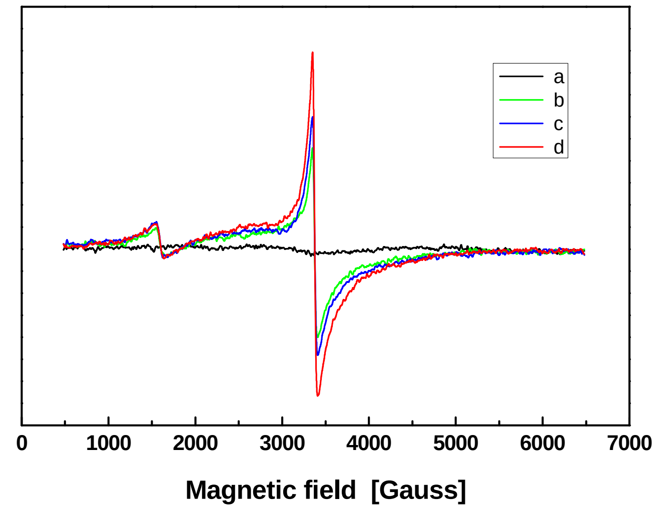

Figure S6. EPR spectra of (a) HBPU-DMPA-DOPA, (b) HBPU-DMPA-[Fe(DOPA)$_3$] complexes, pH = 4, (c) HBPU-DMPA-[Fe(DOPA)$_3$] complexes, pH = 7, and (d) HBPU-DMPA-[Fe(DOPA)$_3$] complexes, pH = 9.

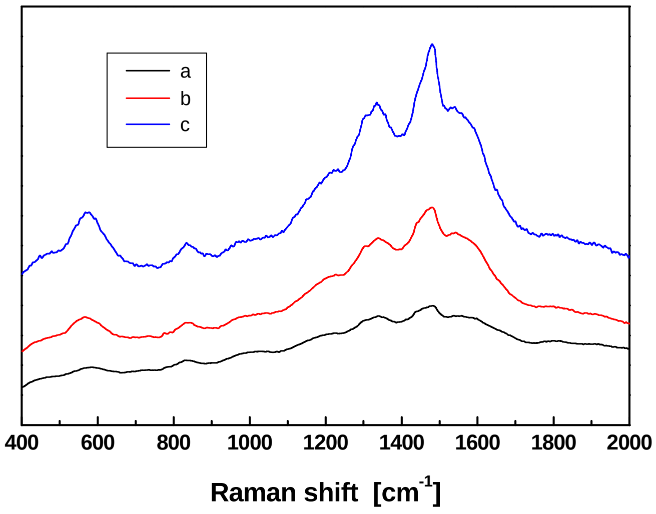

Figure S7. Raman spectra of HBPU-DMPA-[Fe(DOPA)$_3$] complexes. (a) pH = 4 (black curve), (b) pH = 7 (red curve), and (c) pH = 9 (blue curve).

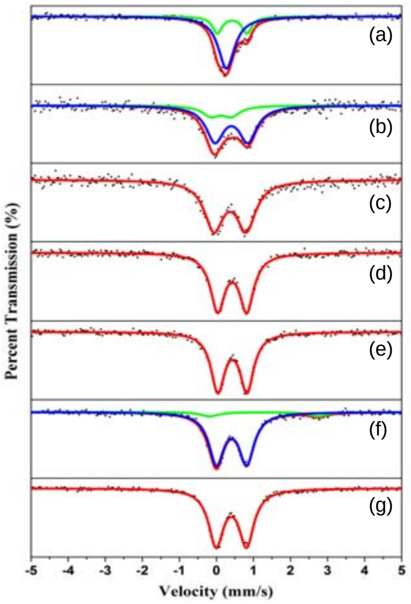

Figure S8. Room temperature ^{57}Fe Mössbauer spectra of (a) anhydrous ferric chloride, (b) Fe[DOPA]$_3$ (pH = 9), (c) water saturated Fe[DOPA]$_3$ (pH = 9), (d) Fe[DOPA]$_2$ (pH = 7), (e) water saturated Fe[DOPA]$_2$ (pH = 7), (f) Fe[DOPA] (pH = 4), and (g) water saturated Fe[DOPA] (pH=4).

The data in **Figure S8** and **Table 1** show that under the condition of pH = 9 the Mössbauer parameters of Fe[DOPA]$_3$ are quite different for the anhydrous and water saturated samples. In the case of anhydrous sample, its Mössbauer spectrum exhibits two types of signals suggesting the presence of two states of iron. When the sample is saturated by water, however, only single state of iron appears. These evidence that tris coordination between DOPA and Fe^{3+} acts as the main role in DOPA-iron interaction in the water saturated Fe[DOPA]$_3$ and the dynamic coordination-dissociation of DOPA-iron interaction leads to the single state of the iron. As long as water is removed, the dynamic coordination is frozen so that tris and bis coordination bonds are detected, reflecting the transient behavior of the DOPA-iron interaction. That is, although tris coordination bonds predominate in the anhydrous Fe[DOPA]$_3$ (relative component area = 78 %), there are also a few bis coordination bonds (relative component area = 22 %). The symmetry of bis-coordinated iron is slightly increased in this case as revealed by the low QS of 0.55 mm s^{-1}, which is smaller than that of the tris-coordinated iron (0.89 mm s^{-1}) (refer to Y. Yoshida, G. Langouche, Mössbauer Spectroscopy, Springer, Berlin, 2013, p.121).

For pH = 7, the Mössbauer parameters of the anhydrous $Fe[DOPA]_2$ resemble those of the water saturated version, and only single state of iron appears in both the samples. Clearly, the dynamic coordination of iron ions in the water saturated sample behaves like the static coordination in the anhydrous sample. Considering that (i) the two types of samples have the same IS and QS (0.43 and 0.78 mm s^{-1}), (ii) the IS values are greater than those of the samples at pH = 9, and (iii) the QS values are lower than those of the samples at pH = 9, we know that resonated bis coordination between DOPA and Fe^{3+} must be the major form of DOPA-iron interaction no matter whether water is present or not. The deduction is further supported by the fact that O-Fe bond length increases with decreasing coordination number, and bis coordination leads to higher symmetry of the distribution of extranuclear electron cloud of iron ions than that of tris coordination.

When pH is reduced to 4, two types of resonance signals are observed again on the Mössbauer spectrum of the anhydrous Fe[DOPA], and single type of signals is perceived in the water saturated sample. In this case, bis- and mono-coordination govern the DOPA-iron interaction. The Mössbauer parameters of the mono-coordinated iron are IS = 1.29 mm s^{-1} and QS = 2.93 mm s^{-1}. Such an asymmetric and unsaturated structure would interfere with the bis-coordination between DOPA and Fe^{3+}. Consequently, the bis-coordinated iron in the anhydrous sample exhibits slightly lower IS but higher QS than the case at pH = 7. On the other hand, the higher IS of the mono-coordinated iron is attributed to an electron transfer from the dopamine molecule to the octahedral Fe sites (refer to J. Fouineau, K. Brymora, L. Ourry, F. Mammeri, N. Yaacoub, F. Calvayrac, S. Ammar-Merah, J.-M. Greneche, Synthesis, Mössbauer characterization, and ab initio modeling of iron oxide nanoparticles of medical interest functionalized by dopamine, J. Phys. Chem. C, 2013, 117, 14295). When the sample is saturated by water, single state of iron is perceived because of the dynamic nature of the coordination bonds.

In summary, two states of iron are always observed in the anhydrous samples, while single state of iron is observed in water saturated samples. This proves that the DOPA-iron complexation becomes dynamic in the presence of water and the dynamic manner is immobilized after removing water. Because of the highly symmetric structure and resonance of bis-coordination, the samples at pH = 7 is an exception. Even the dynamic coordination is fixed in the anhydrous sample, the dynamic feature

is still displayed so that similar state of iron is found in both water saturated and anhydrous samples.

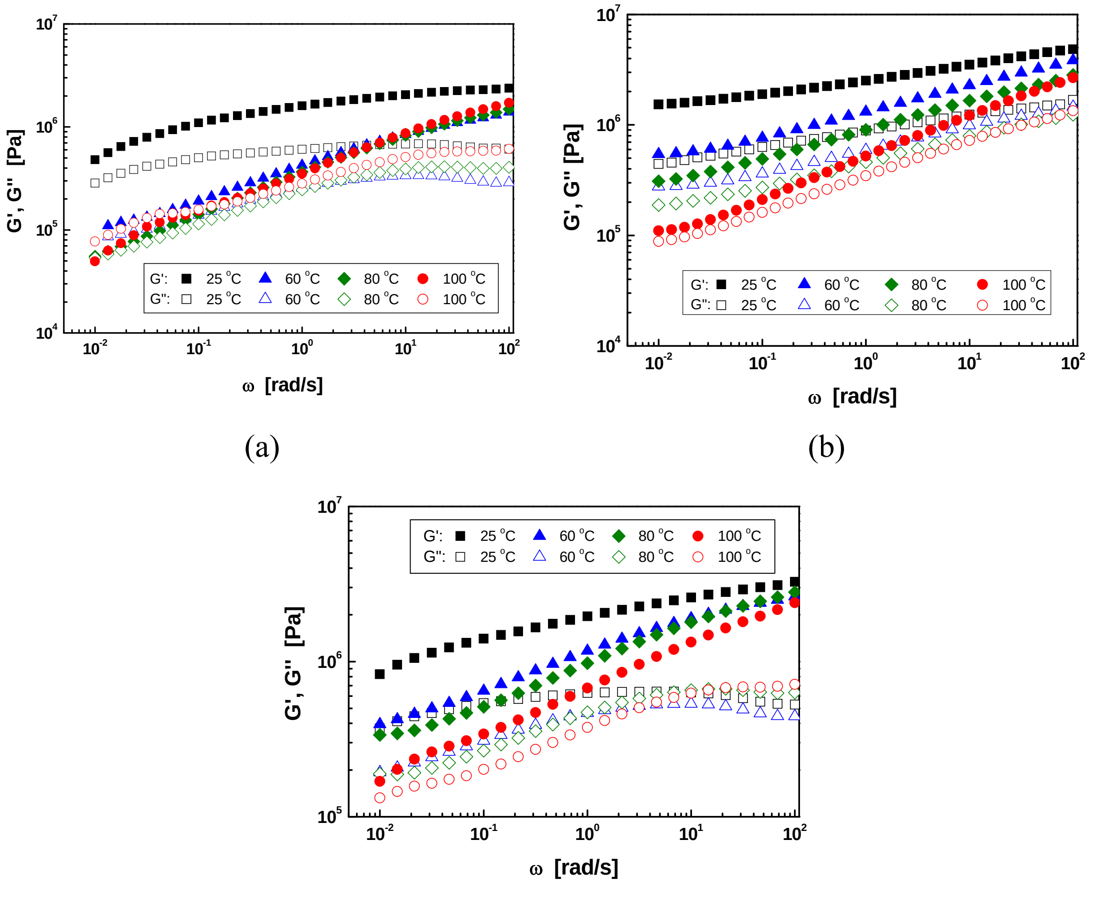

(c)

Figure S9. Storage shear modulus, G′, and loss shear modulus, G″, as a functions of oscillatory frequency, ω, for (a) HBPU-DMPA-[Fe(DOPA)$_3$] saturated by water at pH = 9, (b) HBPU-DMPA-[Fe(DOPA)$_3$] that was firstly saturated by water at pH = 9 and then dried, and (c) HBPU-DMPA-DOPA saturated by water at pH = 9.

Figure S9a shows that G’ is higher than G” in most cases and both G’ and G” decrease with decreasing frequency. Nevertheless, the two curves intersect at certain frequency under 80 and 100 °C, meaning that the rheological behavior of the specimen is changed from elastic-like behavior (G’ > G”) to viscous-like behavior (G’ < G”) at low frequency regime. This transition is indicative of dynamic coordination-dissociation of catechol-Fe^{3+} crosslinks. At certain low frequencies (for a

given temperature), the dynamic bonds start to exert significant influence on rheological performance of the polymer because the disconnected network needs time to be reconstructed. Besides, the crossover frequency between G’ and G” increases with increasing temperature. Because this frequency is related to the dissociation rate constant of the reversible network (refer to M. C. Roberts, M. C. Hanson, A. P. Massey, E. A. Karren, P. F. Kiser, Dynamically restructuring hydrogel networks formed with reversible covalent crosslinks, Adv. Mater. 2007, 19, 2503), it implies that the dynamic catechol-Fe^{3+}complexation is accelerated with a rise in temperature. This is in agreement with the general behavior of reversible reactions.

In the case of dried HBPU-DMPA-[Fe(DOPA)$_3$] (**Figure S9b**), the frequency dependence of G’ does not intersect with that of G” within the temperature range of interests. It means that the dynamic catechol-Fe^{3+}complexation is not activated in the absence of water.

AS for HBPU-DMPA-DOPA (**Figure S9c**), which does not contain any catechol-Fe^{3+} coordination bonds, the material behaves like other conventional polymers and G’ > G” within the frequency range of interests.

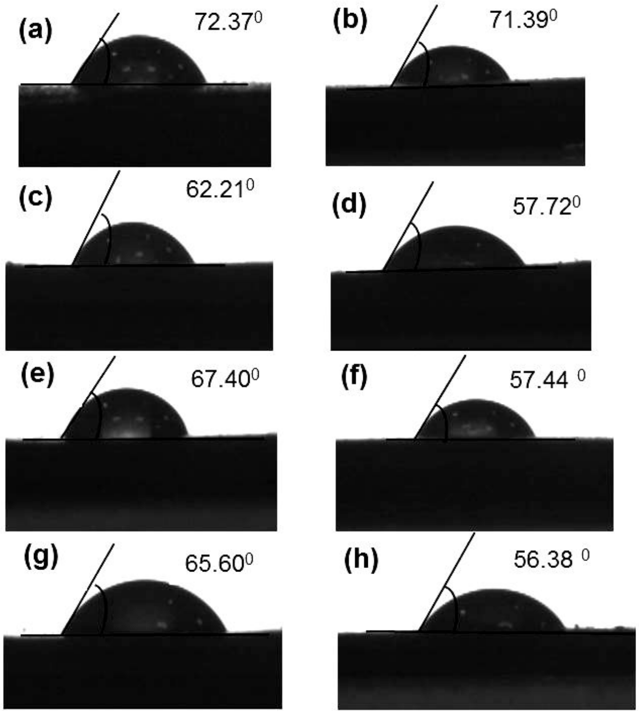

Figure S10. Water contact angles of (a) molded surface of HBPU-[Fe(DOPA)$_3$], (b) cut surface of HBPU-[Fe(DOPA)$_3$], (c) molded surface of HBPU-DMPA-[Fe(DOPA)$_3$], (d) cut surface of HBPU-DMPA-[Fe(DOPA)$_3$], (e) molded surface of HBPU-DMPA-DOPA, (f) cut surface of HBPU-DMPA-DOPA, (g) molded surface of HBPU-DMPA-phenethylamine, and (h) cut surface of HBPU-DMPA-phenethylamine.. Here, HBPU-[Fe(DOPA)$_3$] serves as the reference material. The difference between HBPU-[Fe(DOPA)$_3$] and HBPU-DMPA-[Fe(DOPA)$_3$] lies in the fact that the former was not hydrophilically modified by DMPA.

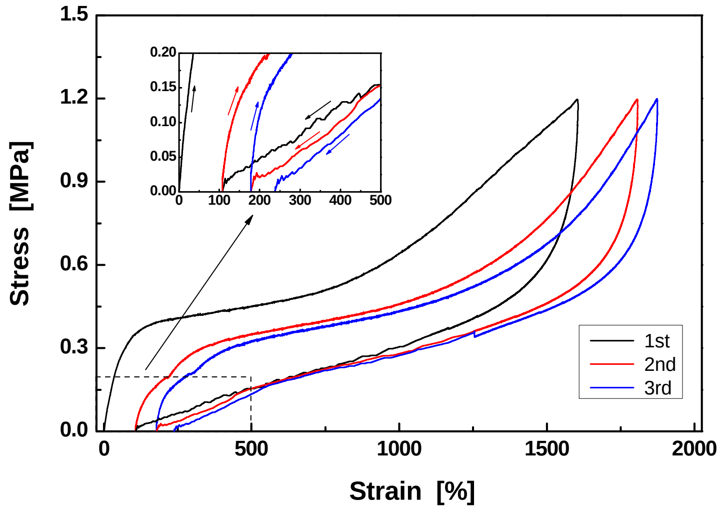

Figure S11. Tensile stress-strain curves recorded during cyclic loading and unloading tests of HBPU-DMPA-DOPA in water (pH = 9) at 25 °C. Arrows indicate directions of loading and unloading. Only cycles 1, 2 and 3 are shown for clarity (cycles of 4 and 5 are not given). The cyclic tests were continuously conducted without resting between successive cycles.

The cyclic tensile stress-strain curves were measured beyond the yield region. In the case of HBPU-DMPA-[Fe(DOPA)$_3$] (**Figure 2b**), except for the first hysteresis loop due to permanent plastic deformation resulting from forced movement of chain segments and destruction of partial weak irreversible molecular interaction, the subsequent hysteresis loops measured almost overlap each other. More importantly, no residual strain is detected after the second and third cycles. These closed hysteresis loops should be related to the dynamic bonding of catechol-Fe^{3+} complexation, which helps to rapidly re-establish and rearrange the crosslinks in the course of unloading. Comparatively, residual strains are found after the second and third cycles in HBPU-DMPA-DOPA and the corresponding hysteresis loops do not overlap (**Figure S11**), except that the appearances of the curves look to be similar to those of HBPU-DMPA-[Fe(DOPA)$_3$]. It means that HBPU-DMPA-DOPA cannot be completely recovered like HBPU-DMPA-[Fe(DOPA)$_3$] due to lack of the dynamic bonds.

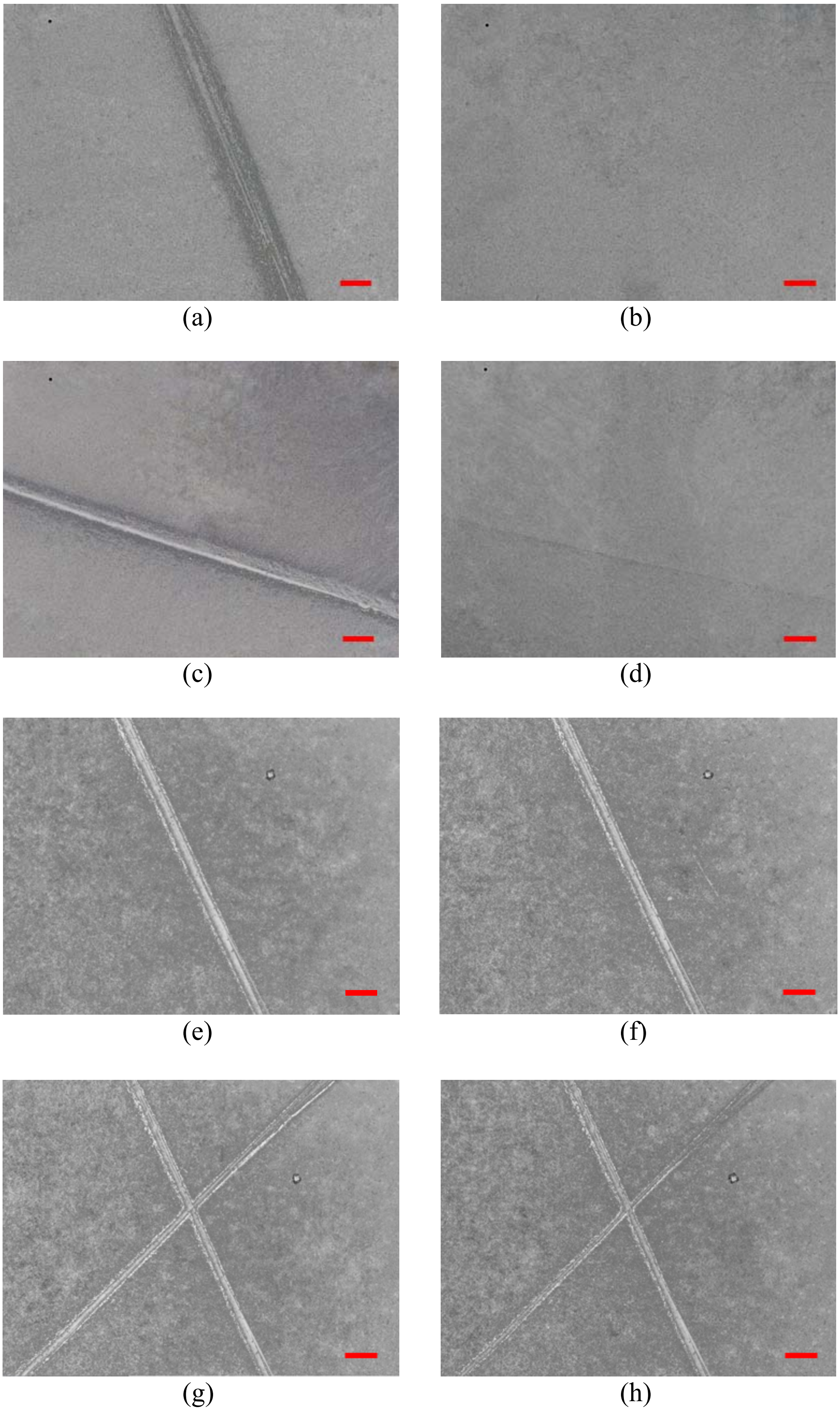

Figure S12. Optical images showing self-healing of razor cuts on (a-d) HBPU-DMPA-[Fe(DOPA)$_3$] and (e-h) the control HBPU-DMPA-DOPA. The scale bars represent 50 μm in length. (a, e) The first cut. (b, f) Effect of the first healing. (c,

g) The second cut. (d, h) Effect of the second healing. Both the cut and healing were conducted in artificial seawater at 25 °C. Healing time: 24 h. Because the swelling rates of HBPU-DMPA-[Fe(DOPA)$_3$] and HBPU-DMPA-DOPA are similar (refer to **Figure S13** shown below), especially at 24 h (i.e. the time of self-healing), and their polarities are also similar (refer to **Figure S10**), the observed crack disappearance should not be due to differences in polymer swelling between the two samples.

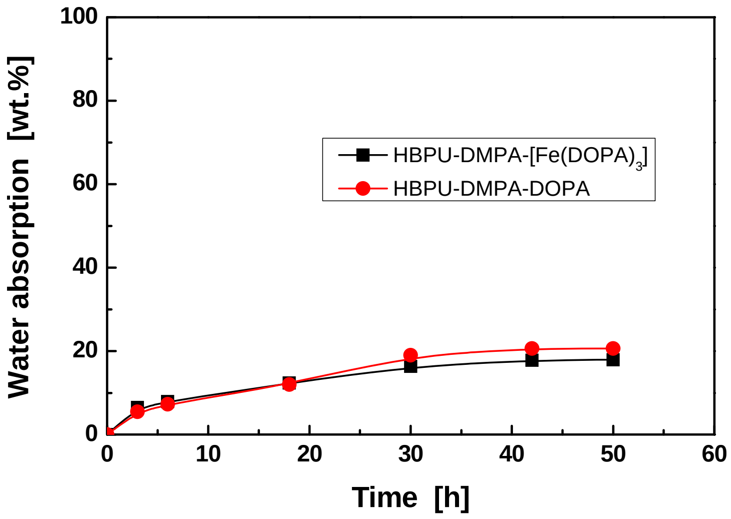

Figure S13. Water absorption versus time of HBPU-DMPA-[Fe(DOPA)$_3$] and HBPU-DMPA-DOPA in artificial seawater at 25 °C.

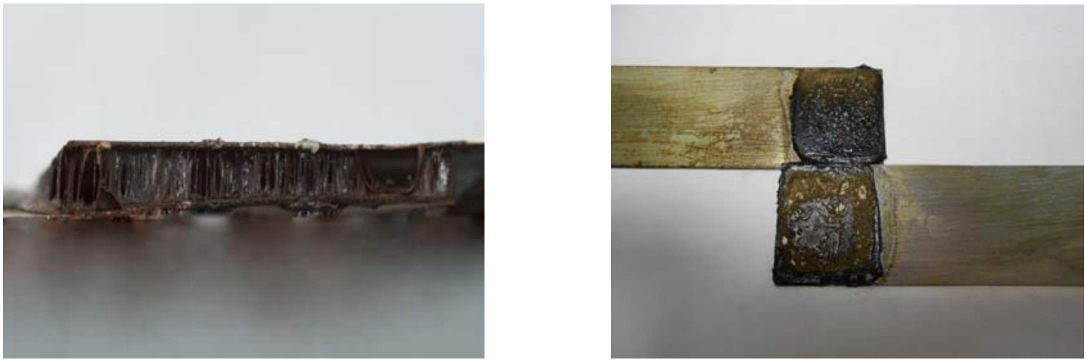

(a) (b)

Figure S14. Lap shear test of iron plates bonded by HBPU-DMPA-[Fe(DOPA)$_3$] in artificial seawater at 25 °C. (a) The deformed HBPU-DMPA-[Fe(DOPA)$_3$] between the two iron plates. (b) The split iron plates showing residual polymer sticking to the opposite sides of the iron plates due to cohesive failure. Although the lap shear test led to less severe deformation of the material than tensile test, the fractured surfaces were still uneven, which cannot be flattened by the mild pressure of 0.15 MPa applied for keeping apparent contact between the broken specimens during healing. With increasing number of tests, more and more non-contact regions appear, which reduces the effective healing at the interface so that the measured shear strength decreases accordingly (refer to **Figure 3c**). However, the phenomenon does not perfectly reflect the nature of the material. The AFM pull-off force measurement demonstrates that HBPU-DMPA-[Fe(DOPA)$_3$] indeed possesses repeated self-healability (refer to **Figure 3d**).

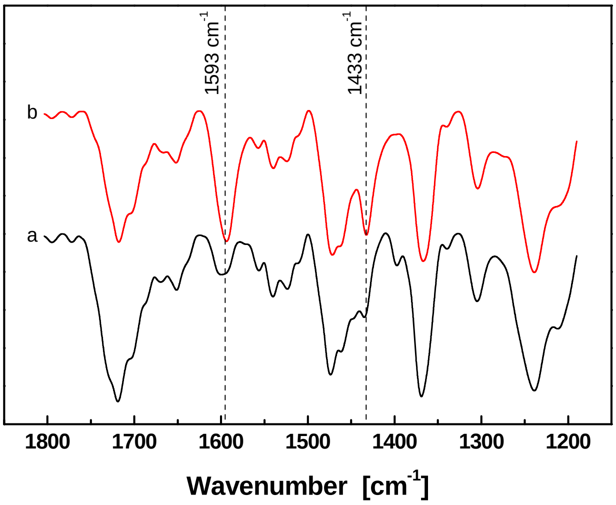

Figure S15. Attenuated total reflectance infrared spectra of (a) molded surface and (b) cut surface of HBPU-DMPA-[Fe(DOPA)$_3$]. The peaks at 1433 and 1593 cm^{-1}, which are attributed to the symmetrical stretching and antisymmetric stretching of carboxyl groups, are obviously higher on the spectrum of the cut surface than those on the spectrum of the molded surface. It means that more carboxyl groups are exposed on the cut surface. The difference might originate from the fact that when the specimen was made in silicone mold by casting, the hydrophilic carboxyl groups tend to hide beneath the specimen surface during the solvent evaporation. Accordingly, there are less carboxyl groups on the molded surface of the specimen.

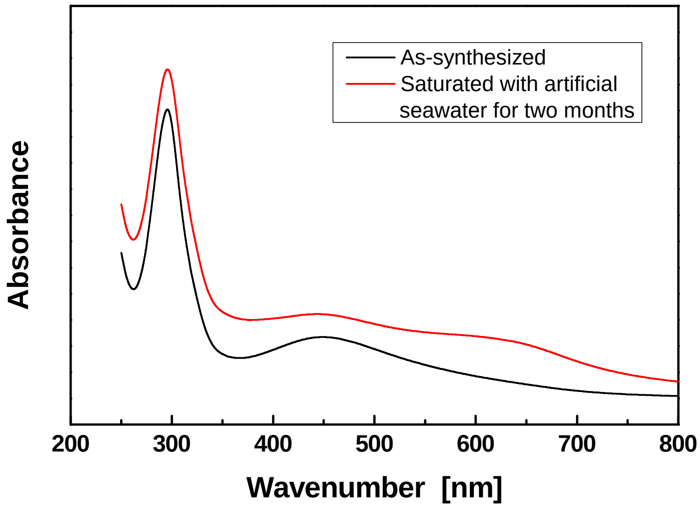

Figure S16. UV-vis spectrum of HBPU-DMPA-[Fe(DOPA)$_3$] that was kept being saturated with artificial seawater and exposed to air for two months at 25 °C in comparison with that of the as-synthesized version.

The absorbance maximum at ~280 nm is the characteristic absorption peak of dopamine, while the peak at 400~500 nm represents the formation of Fe[DOPA]$_3$ in the polymer (refer to N. Holten-Andersen, M. J. Harrington, H. Birkedal, B. P. Leed, P. B. Messersmith, K. Y. C. Lee, J. H. Waite, pH-induced metal-ligand cross-links inspired by mussel yield self-healing polymer networks with near-covalent elastic moduli, PNAS, 2011, 108(7): 2651). The characteristic absorption peak of dopamine quinone at ~400 nm does not appear on the two spectra, suggesting that dopamine was not oxidized during either synthesis or service in seawater.

.

Table S1. AFM peak pull-off force of different polymers

Polymers	Specification	Average peak pull-off force* (nN)
HBPU-DMPA-[Fe(DOPA)$_3$]	Saturated by water, pH=9	-40.4
	Saturated by water, pH=7	-37.2
	Saturated by water, pH=4	-34.1
HBPU-DMPA-DOPA	Saturated by water, pH=9	-12.4
	Saturated by water, pH=7	-11.5
	Saturated by water, pH=4	-12.9

*The negative signs are only indicative of movement direction of the probes.

Although HBPU-DMPA-DOPA also exhibits similar behavior in the course of AFM pull-off force measurement due to hydrogen bonding, its peak pull-off force that represents the maximum interaction between the same material is much lower than that of HBPU-DMPA-[Fe(DOPA)$_3$] (refer to **Figure 3d** and **Table S1**). Additionally, the peak pull-off forces of HBPU-DMPA-[Fe(DOPA)$_3$] decrease with decreasing pH, while those of HBPU-DMPA-DOPA nearly do not change with pH (refer to **Table S1**). The results well agree with the characteristics of catechol-Fe^{3+} complexation and hydrogen bonding, respectively.
